# Assessing the Efficacy of Laser Therapy and Autologous Platelet-Rich Plasma (PRP) Treatment for Chronic Wounds

**DOI:** 10.3390/jpm14010085

**Published:** 2024-01-11

**Authors:** Sandor Mircea, Maghiar Laura, Horgos Maur Sebastian, Borza Ioan Lucian, Farcas Dorina Maria, Ciurescu Daniel, Cheregi Cornel Dragos, Hurjui Liliana Loredana, Sachelarie Liliana, Maghiar Paula Bianca

**Affiliations:** 1 Department of Surgical Disciplines, Faculty of Medicine and Pharmacy, University of Oradea, 410073 Oradea, Romania; drims75@yahoo.com (S.M.); horgos.maur@didactic.uoradea.ro (H.M.S.); badea.paula.bianca@didactic.uoradea.ro (M.P.B.); 2 Preclinical Sciences Department, Faculty of Medicine and Pharmacy, University of Oradea, 1st December Square 10, 410073 Oradea, Romania; lauratodan@yahoo.com; 3 Department of Morfological Disciplines, Faculty of Medicine and Pharmacy, University of Oradea, 410073 Oradea, Romania; ioan.borza@didactic.uoradea.ro; 4 Department of Medical Disciplines, Faculty of Medicine and Pharmacy, University of Oradea, 410073 Oradea, Romania; dmfarcas@yahoo.com; 5 Faculty of Medicine, Transilvania University, 500036 Brașov, Romania; daniel.ciurescu@unitbv.ro; 6 Department of Medical Disciplines, Faculty of Medicine and Pharmacy, “Grigore T. Popa” University of Medicine and Pharmacy, 700115 Iasi, Romania; loredanahurjui@gmail.com; 7 Department of Preclinical Disciplines, Faculty of Medicine, Apollonia University, 700511 Iasi, Romania

**Keywords:** wound, laser, PRP (platelet-rich plasma)

## Abstract

(1) Background: The management of persistent wounds is a topic of significant concern, particularly when they become chronic. Clinicians are focused on reducing the healing duration of chronic wounds and employing the most efficient treatments. Successful wound management requires an understanding of standard healing processes, the elements that can impede healing progress, and strategies to manage these impediments. (2) Methods: We participated in a study involving a cohort of 115 patients. Data were collected through subjective assessments via questionnaires, examining the comparative effects of laser therapy and platelet-rich plasma (PRP) on patients’ conditions and monitored the progression of chronic wounds. For the study, we utilized a branded laser equipped with a 12-watt probe to stimulate chronic wounds in 65 patients, while the PRP procedure was administered to 50 patients. (3) Results: We observed a greater improvement in local symptoms among the patients who received laser therapy compared to those in the PRP group. (4) Conclusions: We consider both therapies to be of significant importance due to their positive and beneficial effects, particularly on the symptomatology and progression of chronic wounds. Nevertheless, superior results were documented in patients who underwent laser therapy.

## 1. Introduction

Each wound necessitates a customized treatment plan, taking into account factors such as its etiology, chronicity, anatomical location, degree of microbial contamination, and patient-specific variables influencing the healing process [1]. Incorrect dressing application can impede the healing trajectory, and chronic wounds can significantly impact a patient’s quality of life. The consequences of wounds encompass pain, distress, social seclusion, anxiety, prolonged hospitalizations, persistent illnesses, and even mortality, many of which can be preventable. Additionally, certain chronic wounds exhibit atypical healing patterns due to underlying factors such as patient age or concurrent chronic comorbidities. These “challenging-to-heal” chronic wounds, defined as those that are unresponsive to conventional care, further diminish the patient’s quality of life and exert an escalating burden on the healthcare system over time [2,3].

Wound healing is no longer constrained to the classical phases of inflammation, proliferation, and maturation; it relies on intricate interactions between cells and mediators facilitating the process [3]. On a macroscopic scale, wound healing is influenced by diverse factors, including wound size, depth, anatomical location, patient age, involvement of a specialist, and systemic health status. Several factors contribute to suboptimal wound healing, such as reduced oxygen supply, persistent inflammation, age-related fibroblast dysfunction, alterations in critical cytokines, growth factors, receptor concentrations, and the presence of infections [4,5,6]. It is imperative to explore innovative interventions that are aimed at expediting the healing process of chronic wounds. This approach can yield a dual benefit: reducing patient costs associated with home care and hospitalization, while mitigating hospital admissions. Concurrently, restoring regular patient life and promoting early mobility and social reintegration are essential goals [4,5,6,7,8]. Psychological motivation and a swift return to normalcy are advantageous, emphasizing the importance of treatments that are minimally invasive and gentle on both the overall and local patient well-being [8,9,10].

Laser therapy involves the application of infrared light to specific areas that are affected by a range of conditions, with the aim of enhancing soft tissue healing, benefiting both acute and chronic ailments [11]. High-intensity laser therapy, in particular, augments local microcirculation and aids lymphatic drainage in pathological regions.

By combining biostimulation and photomechanical stimulation, laser therapy not only offers a potent approach to pain management but also actively contributes to tissue repair. The high-intensity laser system presents highly efficacious treatment modalities for a diverse array of clinical indications [11,12,13].

Incorporating platelet-rich plasma (PRP) can enhance the effectiveness of various regenerative medicine treatment approaches, making it a valuable complementary tool in this field. Platelets, unique blood components, are pivotal in initiating both hemostasis and the healing process [8]. PRP therapy operates by releasing growth factors from platelets through alpha granule degranulation. These alpha granules contain growth factors like PDGF (platelet-derived growth factor), EGF (epidermal growth factor), and VEGF (vascular endothelial growth factor), which hold essential roles in the wound healing process. These growth factors bind to transmembrane receptors on the surfaces of epidermal cells, fibroblasts, osteoblasts, and endothelial cells, instigating the wound healing cascade. This leads to fibroblast proliferation, extracellular matrix formation, and increased collagen synthesis. PRP also possesses the potential to mitigate inflammation at the wound site, thus facilitating the healing process. Additionally, macrophages are recruited to the wound site, contributing to bactericidal actions that reduce the biological burden on the wound [9].

In our research, we evaluate the results achieved through two innovative treatments for chronic wounds: high-intensity laser therapy and the platelet-rich plasma injection method (PRP technique).

The objective of this current study is to offer insights into the healing process by comparing the application of laser therapy and PRP for chronic wounds.

It is important to note that both laser therapy and PRP should be administered under the supervision of qualified healthcare providers who can assess the patient’s specific wound condition and determine the most appropriate treatment plan. Additionally, these therapies are often used as part of a comprehensive wound care strategy that includes infection control, debridement, and other established wound management techniques.

## 2. Materials and Methods

### 2.1. Aim of the Study

This study is an observational study, and we proposed to highlight the beneficial effect of using the laser and PRP in chronic wounds.

### 2.2. Materials

We conducted a retrospective study, carried out over a period of 18 months, between 1 October 2021 and 31 December 2022. In the study, a total of 115 patients from the Surgery department of CF Oradea Clinical Hospital, who were hospitalized with chronic wounds, were included.

The total patient population was divided into two distinct study groups, each receiving one of two novel therapies. One study group consisted of 65 patients who underwent laser therapy, while the other group included 50 patients who underwent the platelet-rich plasma (PRP) injection procedure. The essential data required for the study encompass information collected from patient observation sheets, including age, gender, place of origin, medical history, and personal and pathological backgrounds, along with responses from the subjective evaluation questionnaire.

### 2.3. Criteria for Selecting Subjects

Patient demographic data, including age, gender, and background, plays a crucial role in the stratification of individuals into distinct cohorts. This stratification facilitates a more comprehensive analysis of the influence of these variables on the development of the medical condition under study. Information about the patients’ medical histories offers valuable insights into the underlying factors that are responsible for the persistence of chronic wounds. Among the most prevalent contributing factors are chronic arteriopathy obliterans, chronic venous insufficiency, and diabetes.

Inclusion criteria encompassed individuals who had not previously undergone alternative treatments for wound healing, those with underlying medical conditions such as diabetes, chronic obstructive arteriopathy, and chronic venous insufficiency associated with varicose ulcers, as well as patients with wounds exhibiting a protracted course.

Exclusion criteria were applied to patients with superinfections of the wounds (demonstrated by cultures indicating negative chronic wounds), chronic wounds resulting from trauma or contusions, and other conditions that could impede their participation in the study. This included individuals with limited cooperation, severe medical conditions significantly impacting their daily life (e.g., cancer, advanced renal or liver failure), and those afflicted by immunodeficiency diseases such as HIV or AIDS.

Exploring the limitations of a study is a critical aspect of research, as it helps researchers and readers understand the scope and reliability of the study’s findings. There are some common limitations that researchers may encounter when studying the application of laser therapy and PRP for chronic wounds, like the small sample size, which may limit the generalizability of the results, and non-randomized studies can have limitations in terms of establishing causation and controlling for confounding variables.

Chronic wounds can vary widely in terms of etiology, location, size, and severity. Studies often involve a diverse group of patients with different types of chronic wounds, which can make it challenging to draw uniform conclusions about the effectiveness of laser therapy or PRP for specific wound types.

Research studies may not always include a diverse range of participants in terms of age, gender, race, and comorbidities, which can limit the generalizability of the findings to a broader population.

Further research and well-designed clinical trials can help mitigate some of these limitations and provide more robust evidence for the efficacy of these therapies.

The number of the patients was selected according to how they applied for treatment, with laser or PRP.

The present study observed the ethical conditions established by the Helsinki Declaration, being approved by the local Ethic Committee of CF Clinical Hospital of Oradea–nr. 55/04.01.2022.

### 2.4. Methods

In the conducted study, we analyzed the questionnaires completed by both groups of patients. We evaluated the primary symptoms, including local pain (measuring pain intensity), nighttime discomfort, walking difficulties, and the overall condition reported on the day of admission.

Each patient voluntarily participated in the study and provided written informed consent. At the time of enrollment, a consent form was signed by each patient. In doing so, each patient acknowledged the study’s objectives, general aspects, and the treatments involved.

In the first study group, laser treatment was administered following wound cleansing, starting from the day of admission and continuing for seven consecutive days of treatment.

In the second study group consisting of 50 patients, platelet-rich plasma (PRP) therapy was employed. PRP is increasingly utilized in the treatment of chronic skin wounds in conjunction with other wound care modalities. PRP contains high concentrations of platelets and growth factors that can promote and support the process of wound healing.

The PRP is prepared using a medical device kit, which includes a test tube containing a separating gel made from an inert polymer and an anticoagulant (sodium citrate 3–4%). Each tube has a precalibrated vacuum designed to collect 10/12 mL of blood. To obtain platelet-rich plasma, the collected blood is subjected to centrifugation at 4000 rpm for 5 min. After centrifugation, the test tube will have a clear yellowish layer at the top, followed by a separation gel barrier and a red bottom layer. The next step involves removing the test tube from the centrifuge and gently mixing the plasma and platelets on top of the gel by exposing it to a half-turn motion.

The PRP formulation is extracted using a transfer device. It is then injected onto the wound bed, maintaining a distance of approximately 2 mm from the wound’s periphery towards the center. The serum was administered on the 5th day of hospitalization, after the necessary preparations were made to ensure the optimal timing for injection.

### 2.5. Statistical Analyses

We analyzed data from our study with SPSS 26, T-Test, to assess the comparative effect of laser treatment and PRP on the study groups with statistical significance for <0.001.

## 3. Results

Upon analyzing the data and comparing the two study groups based on gender, we observed that female patients predominate. Specifically, the female population comprises 61.5% of the first group and 60% of the second group. Regarding the male patients, they accounts for 38.5% in the first group and 40% in the second group. This distribution demonstrates an effort to maintain a balanced gender ratio between the two study groups.

It is important to note that there is a notable difference between the two groups in terms of their places of origin. In each individual study, we conducted a natural selection of patients and did not aim to achieve a specific target. Therefore, we can classify this study as a randomized one, as indicated in Table 1.

When comparing the results between the two study groups, we observe that the most common underlying disease associated with chronic wounds is Chronic Obliterating Arteriopathy (ACOMI) of the lower limbs, with 33 cases in the first group and 25 cases in the second group. Diabetes is the second most common underlying disease, with 18 cases in the first group and 14 cases in the second group. Chronic venous insufficiency is the least common in our research, with 14 cases in the first group and 11 cases in the second group, where the PRP procedure was performed.

From a percentage perspective, we note a balanced distribution of these pathologies between the studied groups. The percentage difference is only 4% among patients with ACOMI, 1% among patients with Diabetes Mellitus, and 1% among those with Chronic Venous Insufficiency, as shown in Table 1. Thus, the disparities in the prevalence of these three pathologies are relatively minor, as indicated in Table 1.

The data were statistically analyzed using SPSS 24. Statistical significance was considered at *p* < 0.01.

There was no statistically significant difference between the demographic characteristics of the laser group and PRP group(*p* > 0.001), and the size of the chronic wounds in the two study groups is statistically insignificant (*p* > 0.01).

The underlying pathologies sometimes impact the mobility of patients. The mobility scale used in this study ranges from 0 to 5, with 5 indicating an inability to walk with the affected limb, and 0 indicating that walking is not affected. When comparing the evolution of the mobility scale between the two groups, it became evident that the laser therapy group showed a quicker improvement in symptoms compared to the PRP group. Upon examining the values of the mobility scale on the day of discharge, we can observe a decrease in values in both groups. However, this decline was more rapid in the laser therapy group, and it continued until the day of discharge. This clinical observation is consistent with the faster improvement in symptoms seen in the laser therapy group, as outlined in Table 2.

**Figure 1:**
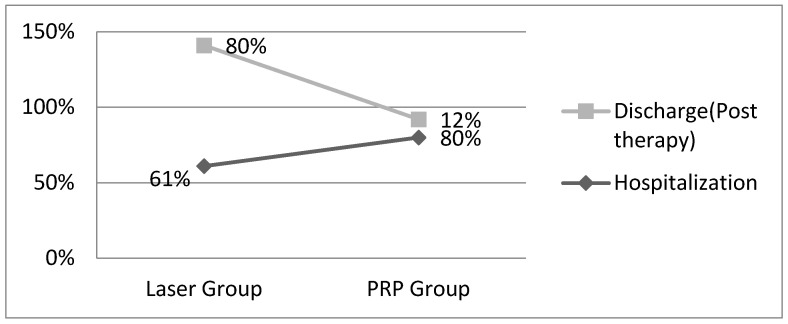
The evolution of pain from the wound for the two groups.

Referring to the day of discharge, a significant disparity in the impact on local pain is evident. The data we have collected clearly indicate that laser therapy has a markedly positive effect on local pain, exerting a robust analgesic influence compared to the PRP procedure. Specifically, on the same day, during laser therapy, 80% of the patients reported pain scores falling within the range of 2 to 4 points, while only 12% reported the same pain score range following the PRP injection. This substantial contrast underscores the superior pain management that is provided by laser therapy in this context (Figure 1).

The nocturnal discomfort experienced by patients during their hospitalization was significantly reduced with the applied treatment. In the first group, 36% of the patients reported that this discomfort had completely disappeared, allowing them to rest without interruptions in their sleep caused by chronic wounds. In contrast, in the second group of patients, only 30% reported the disappearance of this discomfort. By comparing these data, it becomes evident that laser therapy has a notably superior impact on reducing nighttime discomfort when compared to PRP therapy. Upon analyzing the data following the therapies administered at discharge, it is noted that the difference in the size of chronic wounds is not statistically significant (*p* > 0.01). However, when making a comparative assessment, it becomes evident that patients who underwent laser therapy had smaller chronic wounds, with an average size of 2.04 ± 0.29. These findings suggest that laser therapy may have a more favorable effect on chronic wounds compared to PRP, as indicated in Table 3.

The objective results related to the local progression of the wounds were assessed during patient follow-ups. For those who received laser therapy, this evaluation occurred 4 weeks after discharge, whereas it took place 3 weeks after discharge for patients treated with platelet-rich plasma.

Corroborating the obtained data, we mention the following: 30% of the patients in the group that benefited from the laser presented themselves in the wound healing phase, while 14% presented themselves to be in the same phase in the group that was injected with plasma rich in platelets. Regarding the evolution over time and the appearance of possible local complications, we notice that in the laser therapy group, we have a more beneficial effect, as here, only 6% of patients returned with the wound in a stationary or aggravated phase, while in the group with platelet-rich plasma, it was about a percentage of 10%.

**Figure 2:**
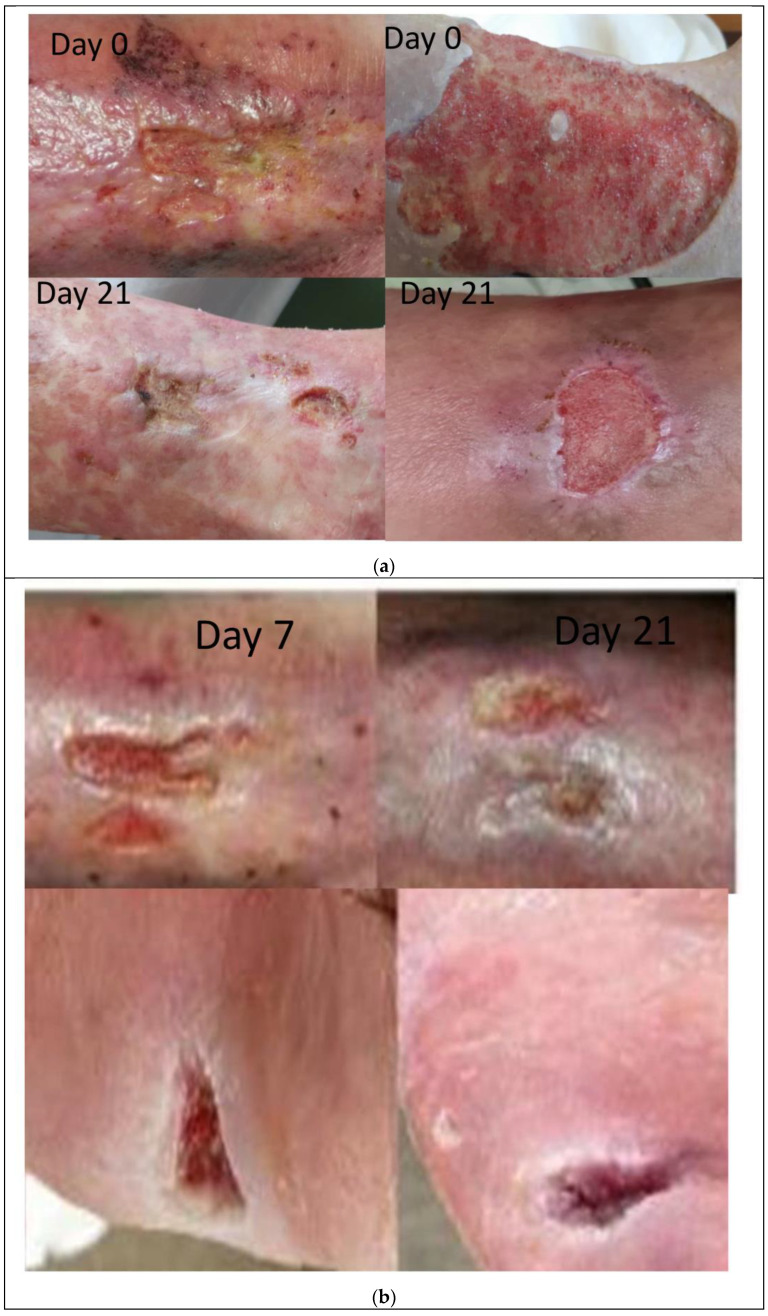
(**a**) Evolutionary aspects before and after laser therapy; (**b**) Evolutionary aspects before and after PRP therapy.

The local evolution of the wound was particularly favorable in the patients who received the laser compared to the patients who were injected with plasma that was rich in platelets. The size of the wound was considerably reduced in most patients following both therapies, see Figure 2.

After evaluating the results, it can be concluded that both therapies offer substantial benefits to patients by expediting the healing process of chronic wounds, each with its unique mode of action.

In particular, laser therapy stands out as especially advantageous for patients experiencing pronounced local symptoms. The high-intensity laser stimulation significantly reduces local pain and diminishes inflammation by promoting local circulation through the heat generated and the stimulation of vasodilation.

On the other hand, platelet-rich plasma (PRP) injection therapy proves more beneficial for patients with less severe local symptoms. This treatment is advantageous because it requires less accommodation and has a lower psychological impact compared to laser therapy, which can stimulate nerve fibers and induce pain through local heating. Furthermore, PRP therapy is noted for its long-lasting benefits and sustained effectiveness over time.

In summary, both laser therapy and PRP injection therapy provide valuable options for patients dealing with chronic wounds. The choice between these treatments may depend on the severity of local symptoms and the individual preferences and needs of the patient.

## 4. Discussion

In recent years, there has been growing evidence of an increasing number of people living with chronic wounds, significantly impacting their quality of life, particularly when combined with intellectual disabilities that limit daily activities. One consistent and distressing symptom reported by these patients is pain [7,8,9,10]. Chronic pain in this context encompasses both physical and emotional aspects and seldom indicates permanent damage. Since chronic wounds result in a loss of skin integrity and damage to nerve fibers, the resulting pain is a combination of nociceptive pain and neuropathic pain caused by nerve damage [14,15].

Complications, such as infections, can significantly impede the healing process, affect a patient’s quality of life, and even raise the risk of sepsis-related mortality. In the case of surgical interventions, infections have a substantial impact on resources, requiring additional staff time, various supplementary procedures, medications, and materials to manage wound complications and their potential consequences [14]. Reducing future wound care costs is crucial, and one effective approach involves influencing the number of chronic wounds that require treatment. This highlights the significant role that injury prevention plays in addressing demographic trends that impact injury prevalence. For instance, there are recommendations to use graduated below-the-knee compression stockings to prevent the recurrence of venous ulcers in patients with healed ulcers. Additionally, regular leg examinations are advised for preventing atherosclerosis, alongside optimizing glycemic control and encouraging smoking cessation [15]. When we consider wound prevention, acute pressure ulcer prevention often takes center stage and is extensively studied. Pressure ulcers have a profound negative impact on various aspects of an individual’s quality of life. Despite not being a new issue, pressure ulcers continue to pose a significant health challenge, especially as the population ages [16].

Effective pain management is rooted in fundamental principles and should commence with an evaluation of wound-related pain and its underlying causes. Achieving the optimal balance between wound and pain management necessitates open communication of this information and shared decision making between the physician and patient. Pain management typically starts with non-drug and pharmacological treatments. Pharmacotherapy, which encompasses the use of local and systemic treatments, can be employed to alleviate the intensity of both persistent and temporary pain. Non-pharmacological therapy encompasses a range of strategies that can be employed at home to mitigate the impact of pain and support the treatment of chronic wounds [17,18].

Before initiating treatment for patients with chronic wounds, the underlying factors that are involved should be diagnosed and treated whenever possible. Wound healing can be promoted by topical care [19]. Debridement, or at least wound irrigation, is often required before treatment begins. In addition to necrotic areas, fibrin, eschar, or bandage residues must also be removed. Ringer’s solution or saline are the cleaning agents of choice for cleaning the wound when changing dressings. Preservative-free solutions should be used immediately [19,20,21,22].

An essential prerequisite for effective wound healing is the prevention or elimination of clinically significant wound infections. It is important to recognize that nearly every chronic wound is contaminated or colonized with microorganisms, typically without causing clinical issues. Therefore, diagnosing a wound infection from a clinical perspective should rely on identifying local signs of inflammation [20]. In cases where systemic infection is suspected, a complete blood count should be conducted, and additional diagnostic criteria include the presence of fever and chills. Systemic antibiotic therapy should generally be reserved for situations involving septic conditions or generalized sepsis, with only a few exceptions.

In conditions involving injuries, it is advisable to collect wound secretions for culture and antibiogram testing whenever feasible [22].

In our research study, we conducted a comparative analysis of the outcomes achieved through high-intensity laser treatment and platelet-rich plasma injection (PRP technique) for chronic wounds. High-intensity laser therapy induces significant alterations in the blood flow, enhancing the properties of red blood cells and platelets, which may have broader effects beyond mere vasodilation. Consequently, high-intensity laser therapy (HILT) could serve as a valuable supplementary treatment option for patients with microcirculatory disorders [23,24,25,26].

High-intensity laser therapy promotes increased tissue regeneration and repair, reduces inflammation, alleviates pain, and mitigates oxidative stress. It also stimulates mitochondria, leading to heightened adenosine triphosphate (ATP) production and the subsequent release of growth factors. The binding of these growth factors to cell surface receptors triggers signaling pathways that transmit signals to the cell nucleus, promoting gene transcription and thereby enhancing cell proliferation, viability, and migration across various cell types, including stem cells and fibroblasts [24,25,26].

Laser therapy can increase cellular energy production in the form of adenosine triphosphate (ATP). ATP is essential for cellular function and helps cells perform various tasks that are required for healing, promoting the proliferation of fibroblasts and keratinocytes, which are crucial for wound healing. Laser therapy can also stimulate angiogenesis, the formation of new blood vessels, which enhances blood flow to the wound area, delivering oxygen and nutrients that are necessary for healing, has anti-inflammatory effects, reducing the production of inflammatory molecules, and encourages collagen synthesis, which aids in tissue repair and wound closure [11,15]. Laser therapy is a promising adjunctive treatment option in wound therapy that can accelerate the healing process, reduce pain, and improve outcomes for patients with various types of wounds [11].

Autologous platelet-rich plasma (PRP) has gained increasing importance in patient treatment in recent years, with numerous global studies demonstrating its effectiveness for various medical conditions. PRP injections have been extensively researched, with results consistently showing their potential as an antinociceptive (pain-relieving) agent and a promoter of cell proliferation. In the case of intra-articular PRP injections, they have demonstrated the ability to modulate the joint environment, enhance chondrogenesis, and inhibit knee joint degradation, possibly by reducing the production of pro-inflammatory mediators. The therapeutic benefits of PRP can be attributed to the presence of supraphysiological concentrations of biomolecules and growth factors within platelet granules. These substances help maintain homeostasis and stimulate the repair of damaged tissues [27,28,29]. When PRP is applied to a wound or injured tissue, it releases growth factors and cytokines that stimulate tissue repair and regeneration, and these growth factors promote cell proliferation, angiogenesis (formation of new blood vessels), and the production of collagen, a structural protein that is important for wound closure [14].

However, there is a challenge where some growth factors may not be effectively released following PRP injection, potentially diminishing the treatment’s response. To overcome this limitation, bioactive agents that are compatible with the body have been utilized to stimulate platelets, triggering the release of granule contents and leading to the production of platelet-rich growth factor (PRGF). PRGF represents the final product of PRP, devoid of leukocytes and inflammatory cytokines, and contains specific quantities of cytokines and growth factors. This makes PRGF more effective and reduces its associated side effects, such as pain and swelling, in comparison to PRP. It is worth noting that PRGF tends to be more expensive than PRP at present [30,31].

## 5. Conclusions

Based on the results obtained, it is evident that laser therapy has a significantly superior impact in alleviating the dominant symptom, which is local pain, when compared to PRP treatment. However, it is important to note that both of these therapies provide substantial benefits to patients by accelerating the healing process of chronic wounds.

## Figures and Tables

**Table 1:** Characteristics of studied groups.

	Laser Group *n* = 65 Patients (100%)	PRP Group *n* = 50 Patients (100%)	*p* Value
Characteristics			
Age in years, mean (SD)	69.14	63.24	0.9232
Men	25 (38.5%)	26 (40%)	0.9712
Women	40(61.5%)	24(60%)	0.8762
Rural	38 (58.5%)	22 (44%)	0.9113
Urban	27 (41.5%)	28 (56%)	0.8513
Wound characteristics			
Infection	11(16.9%)	15 (30%)	0.0017
No infection	54 (83.7%)	35 (70%)	0.0018
Wound area in cm^2^ median	3.43 ± 0.21	3.54 ± 0.23	0.0411
Wound etiology, n (%)			
Chronic venous insufficiency	14 (21.53%)	11 (22%)	0.3579
II Diabetes Mellitus	18(27.69%)	14 (28%)	0.3721
Chronic Obliterating Arteriopathy	33 (50.7%)	25 (50%)	0.3675

**Table 2:** Comparison of scale mobility on hospitalization and day of discharge.

Scale Mobility	Laser Group MD ± DS	PRP Group MD ± DS	*p* Value
Hospitalization	1.73 1.67	0.33 ± 0.49	<0.001
Discharge	5.64 ± 0.49	2.31 ± 0.83	0.01
P^Laser-PRP^	0.003	0.342	<0.001

**Table 3:** Wound area.

Wound Area in cm^2^ Median	Laser Group MD ± DS	PRP Group MD ± DS	*p* Value
Hospitalization	3.43 ± 0.21	3.54 ± 0.23	0.023
Discharge	2.04 ± 0.29	2.89 ± 0.83	0.231

## Data Availability

The data are available on the request from the corresponding author.
